# Banning Fisheries Discards Abruptly Has a Negative Impact on the Population Dynamics of Charismatic Marine Megafauna

**DOI:** 10.1371/journal.pone.0144543

**Published:** 2015-12-11

**Authors:** Esther N. Fondo, Milani Chaloupka, Johanna J. Heymans, Greg A. Skilleter

**Affiliations:** 1 School of Biological Sciences, University of Queensland, Brisbane, Queensland, Australia; 2 Kenya Marine and Fisheries Research Institute, Mombasa, Kenya; 3 The Scottish Association for Marine Science, Oban, Scotland, United Kingdom; University of Lleida, SPAIN

## Abstract

Food subsidies have the potential to modify ecosystems and affect the provision of goods and services. Predictable Anthropogenic Food Subsidies (PAFS) modify ecosystems by altering ecological processes and food webs. The global concern over the effects of PAFS in ecosystems has led to development of environmental policies aimed at curbing the production or ultimately banning of PAFS. However, the effects of reducing or banning PAFS are not known. We explore the consequences of PAFS removal in a marine ecosystem under two scenarios: 1) gradual reduction, or 2) an abrupt ban, using a mass balance model to test these hypotheses–The reduction or loss of PAFS will: i) modify trophic levels and food webs through effects on foraging by opportunistic species, ii) increase the resilience of opportunistic species to food shortages, and iii) modify predator–prey interactions through shifts in prey consumption. We found that PAFS lower the trophic levels of opportunistic scavengers and increase their food pathways. Scavengers are able to switch prey when PAFS are reduced gradually but they decline when PAFS are abruptly banned. PAFS reduction to a certain minimal level causes a drop in the ecosystem’s stability. We recommend gradual reduction of PAFS to a minimal level that would maintain the ecosystem’s stability and allow species exploiting PAFS to habituate to the food subsidy reduction.

## Introduction

Food subsidies usually present an easy (“ready-made”) and abundant resource that is normally not accessible or available to organisms. Sources of food subsidies in ecosystems are varied and may be from human activities, for example carcasses discarded by game hunters that are fed on by scavengers [1, 2], rubbish dumps that are fed on by various species of predators (dingoes, coyotes and red foxes) [3]; or from natural processes, such as allochthonous dissolved and particulate matter that subsidise lake ecosystems and benefit both benthic and pelagic communities [4], marine carrion and detritus washed on shore providing a trophic base for terrestrial consumers [5], stranded kelp on beaches provides a food source for invertebrate shredders and juvenile fish [6]. Food subsidies in natural ecosystems have the potential to modify ecosystem dynamics [2] causing the decline or the loss of essential goods and services, threatening the world’s life support system [2].

Predictable Anthropogenic Food Subsidies (PAFS) [2] modify ecosystems by altering consumer-resource relationships, simultaneously benefiting several different trophic levels and changing food web structure [1–3, 7]. PAFS have the capacity to influence directly individuals’ fitness, population dynamics and community composition and interactions, resulting in substantial ecosystem modification, with effects pervading adjacent ecosystems [2–3, 5]. In cases where natural food resources are scarce, PAFS may be necessary to supplement energy requirements of some endangered species such as European vultures [8, 9]. Predictability in space and time of subsidised food supplies makes this food resource easier to access compared with many natural food sources [10, 11]. Predictability of food supply may influence, for example, a scavenger’s foraging behaviour and distribution (in time and space), factors important in the survival of both the predator and prey. In cases of environmental stress, predictability plays an important role in the survival of the scavengers [2] and studies have shown the importance of PAFS in improving demographic parameters for the viability of some endangered species [12, 13]. On the other hand, PAFS can have detrimental effects for example, negative density-dependant effects on fecundity of scavenger species [14]. In some cases, scavengers may increase in number and become over-abundant, modifying ecosystems through changes in food webs [2]. PAFS can also cause an ecological trap, where species face increased predation risk from opportunistic carnivores attracted to the feeding stations [15]. Finally, PAFS are considered to have potential as a powerful management tool in conservation and social issues [2], for example, where food is provided voluntarily to improve survival of endangered species, although there are risks associated with these animals then becoming dependent on these food sources [16].

The potential impacts of PAFS on ecosystems globally has led to the development of new environmental policies aimed at curbing the production or, ultimately, banning of PAFS. However, some policy decisions can influence or even contradict biodiversity management and conservation efforts [13]. In Europe, for example, sanitary regulations have drastically reduced carrion available to vultures [7]. The effects of sanitary policies on the demographic parameters of long-lived species such as some endangered European vulture species, was evident during the outbreak of Bovine Spongiform Encephalopathy (BSE) [13, 17]. The vulture population declined due to food scarcity, with the situation worsening in Spain when the policy implementation coincided with the deployment of wind farms within the foraging range of the starved vultures [18].

In marine ecosystems, discards from fisheries represent a major source of PAFS with over 7 million tonnes of catch discarded annually world-wide [19]. The first comprehensive report on by-catch and discards in world fisheries was published in 1983 [20]. Historically, discards were acknowledged as a component of fisheries before the Common Era [21], but have only been recognized as a management problem since the beginning of the 20th Century [22]. Given this long history, the animals in many ecosystems have adapted to this super-abundant source of energy for multiple generations. The sudden removal of such an abundant food source has the potential for marked impacts on a broad range of organisms across the entire food web, including seabirds [23], dolphins [24], sharks [25, 26], amphipods, isopods, cephalopods, ophiuroids, fish [27,28], hermit crab, starfish, whelk and crabs [29, 30]. Internationally, environmental policies are moving towards the banning of discards (e.g. the European Union (EU) Common Fisheries Policy proposed ban on discards, [23, 31, 32]) as part of the global push for greater environmental sustainability of fisheries [23, 31]. The implementation of the discard ban in the EU might still lead to perverse incentives and changes in fishing practices that have not been anticipated and there might be some unknown consequences where the fisheries do not recover but scavengers still suffer from food reduction. However, it is not known how a sudden or even gradual reduction in the availability of PAFS would affect food webs that have long been exposed to the ready food sources. Reducing or banning PAFS could do more harm than benefit to an ecosystem through cascading effects in the food web. In the event of a ban, those species that were previously dependent on PAFS may revert to their original diet, causing a population crash in that prey from sudden increases in predation pressure. An understanding of the effects of reductions in PAFS will assist in making informed decisions on the management of PAFS and conservation of PAFS-dependant species and help to move fisheries to a more sustainable basis.

Even though studies on the impacts of PAFS in ecosystems have been done [2, 5, 33], very few studies have modelled the role of PAFS in marine ecosystems [34–36]. Ecosystem modelling provides an approach to studying the effects of PAFS at very large spatial scales that incorporate natural processes like environmental variability and human activities such as fishing, agriculture etc. Through ecosystem modelling, it is possible to examine the effects of PAFS on ecosystem dynamics and improve our understanding of ecological roles of food and food webs in ecosystems.

In this paper, we explore the consequences of the removal of PAFS from a marine ecosystem under two scenarios, a gradual removal (over 20 years) compared with an abrupt ban on their release into the system. These two scenarios model real world situations where, first, legislation may be introduced to bring in bans on discards slowly over many years (e.g. green zones as part of Marine Park zonation [37]) versus an immediate ban on the release of discards into a region (e.g. the European Union Common Fisheries Policy [23, 31]). The following hypotheses were tested (after [2]):

1. Reduction or loss of PAFS will modify trophic levels and food webs through effects on foraging by opportunistic species. Opportunistic species directly exploit PAFS and also consume species at lower trophic levels that also scavenge on PAFS.
2. Reduction or loss of PAFS will increase the resilience of opportunistic species to food shortages. In cases of food scarcity, PAFS can provide supplementary energy requirements of opportunistic species, thus making them resilient to food shortages.
3. Reduction or loss of PAFS will modify predator–prey interactions through shifts in prey consumption. Reduction of PAFS is expected to increase consumption of prey as food resources decrease.

## Methods

### Study area

We modelled the Moreton Bay ecosystem in south east Queensland, Australia located between 27°01'S—27°50'S and 153°19’E-153°25’E. Moreton Bay is a large, wedge-shaped Bay where prawn trawling has been operating since the 1950s [38]. The prawn catch was estimated to be about 500 tonnes annually [39] with by-catch, which is discarded, of an average 3000 tonnes [39]. (The prawn catch reported over the last two decades is shown in Fig A in S1 File). The discards from prawn trawling in Moreton Bay comprise mainly crustaceans and fish [39] (Table A in S2 File). Discards such as fish are eaten by seabirds and dolphins close to the surface, while the remainder is fed on by pelagic fish and sharks as it sinks down. The major demersal scavengers are sand crabs (family Portunidae) in addition to benthic invertebrates such as hermit crabs, other crustaceans, gastropods and polychaetes [39].

The Moreton Bay Marine Park covering 3400 km^2^, was established in 1993 [40]. In 1997 the Marine park zoning was implemented and in 2007 a review resulted in an increase in the no-take zone from 5% to 16% [40]. The reduction in trawling effort due to the established marine protected area (MPA) has potentially caused a reduction in discards. We took advantage of this large-scale natural experiment to conduct our study and took the opportunity to develop the first trophic mass balance model (in Ecopath with Ecosim) for the area.

### Modelling

A total of eighteen functional groups were selected to represent the Moreton Bay ecosystem, including: seagrass, macroalgae, phytoplankton, discards, detritus, dugongs, green turtles, zooplankton, jellyfish, prawns, macrobenthos, sand crabs, omnivores, demersal fish, pelagic fish, sharks, dolphins and seabirds (Table B in S2 File). The primary producers are represented by phytoplankton, macroalgae and seagrass and form an important part of the trophic structure. Detritus and the discards form the component of excreta and dead biomass. Zooplankton, dugongs and green turtles represent the primary consumers and dugongs and turtles play a key role in structuring the seagrass and algae abundance, distribution and biomass in Moreton Bay [41, 42]. Jellyfish are included as they can respond to changes in environmental conditions by forming blooms [43]. Macrobenthos represent consumers that are important in bioturbation and feed on the remains of discards and detritus. Prawns, sand crabs and fish are important in the fisheries of the Bay. Sharks, seabirds and dolphins represent the top predators in the system.

To model the structure and interactions of Moreton Bay ecosystem, we used a mass balance model in Ecopath with Ecosim (the equations are given in Text A in S2 File). Ecopath trophic models describe the static state of energy flows in a food web and are based upon the model of Polovina [44]. Ecopath with Ecosim models represent complex food web interactions where each functional group of the web may be a species, a group of species or a detritus group [45–47]. The input parameters—Biomass (B), Production to Biomass ratio (P/B), Consumption to Biomass ratio (Q/B) and Ecotrophic Efficiency (EE), were derived from relevant literature and reports on studies done in Moreton Bay, databases and other Ecopath models of similar ecosystems or estimated by the model. We constructed a diet matrix which gives the proportions of food items (sourced from the literature) for each functional group. The input parameters were entered into the Ecopath software to give the outputs: the trophic structure and interactions between the groups.

In Ecopath with Ecosim, the Finn’s Cycling Index (FCI) is the fraction of an ecosystem's throughput that is recycled. This index, developed by Finn [48], is expressed as a percentage, and quantifies one of Odum’s 24 properties of system maturity [49]. The index also strongly correlates with resilience and stability [50]. We used Finn’s Cycling Index as an indicator of the ecosystem’s stability (the ability to maintain structure and integrity). Cycling is considered to be an important indicator of an ecosystem’s ability to maintain its structure and integrity through positive feedback [51], and is used as an indicator of stress [52] and systems maturity [50, 53]. An increase in the FCI would mean the system would recover faster from a perturbation, whereas a system would be expected to take longer to recover from disturbances (lower FCI) when it is in a more degraded state.

To determine the effects of discards on the ecosystem, we constructed 2 Ecopath models of Moreton Bay: one with and one without discards and examined the differences in the food web structure. The tables of input parameters and the diet matrices for these functional groups are given in Tables D and E in S2 File for model with discards (MB 1) and Tables F and G in S2 File for model without discards (MB 2). To examine how discards modify ecosystem dynamics, we used the dynamic Ecosim model in which we included fisheries catch data from the QFish database available at the Queensland Government, Department of Agriculture and Fisheries (http://qfish.daff.qld.gov.au/) to drive and calibrate the model (Table H in S2 File gives the catch data). We calibrated the model using the baseline model alone and then included fishing, with various vulnerabilities and forcing functions on primary producers (see details in Text B in S2 File), to select the best fit for the data. We did a sensitivity analysis of the model using the Monte Carlo routine and examined the model results with differing CVs (100 trials each) for the input parameters. The biomass estimates resulting from the Monte Carlo simulations are given in Table I in S2 File. The resulting biomass ranges were wide with increasing CV for the functional groups but followed the general patterns found in the model.

The two scenarios in the Ecosim model were: Scenario 1- with gradual reduction of discards, which represents the changes that took place in Moreton Bay from 1990 to 2013 (with the changes in discards as shown in Fig A in S1 File) and Scenario 2- ban discards, where the discards were stopped in 1990 by removing discards in the fishery from the model. To compare the changes in biomass of groups between the two scenarios we used the Mann-Whitney U test which is appropriate for non-normal data and robust for small sample sizes. Other hypotheses presented in [2] were not addressed in this study due to limitations of the models and as they require more detailed data on changes in population density, growth rates, and community diversity, information that are mostly lacking for the Moreton Bay system. Another limitation was the absence of catch and effort data to prior 1990 or before the MPA establishment.

## Results and Discussion

### 1) PAFS alter trophic levels and food webs

Fishing discards illustrate how PAFS influence communities and ecosystems, by affecting a range of ecological processes, trophic levels and adjacent ecosystems [2]. Discards are exploited by a large number of organisms, from top predators (seabirds, sharks and dolphins) to invertebrates (such as crustaceans) and covering different zones or habitats (e.g. sea surface, pelagic and benthic) [2]. In the Ecopath models of Moreton Bay, which represent the system’s steady state, opportunistic species (seabirds, dolphins, sharks) exhibited an increase in the number of food pathways (the number of all pathways from primary producers or detritus groups leading to the selected consumer via specified prey) (Table J in S2 File). The number of pathways increased by 6%, 11% and 10% for seabirds, dolphins and sharks respectively, in the presence of discards (Table J in S2 File). In addition, the trophic levels of these groups dropped; with the trophic levels reduced by 10% for both seabirds and dolphins, and 1% for sharks in the presence of discards. These findings support the hypothesis that PAFS alter trophic levels and food webs [2]. The opportunistic species have the potential to exploit PAFS directly, in addition to preying on lower trophic level species that also exploit these PAFS [2], resulting in the lowering of their trophic levels. The lowering of the trophic levels may subsequently result in a reduction of transfer efficiency between the trophic levels; this is of significance especially if the species involved are top predators, as this affects how the energy flows through the ecosystem—and eventually what is produced as “goods” from the ecosystem (production).

### 2) PAFS increase the resilience of opportunistic species

**Fig 1:**
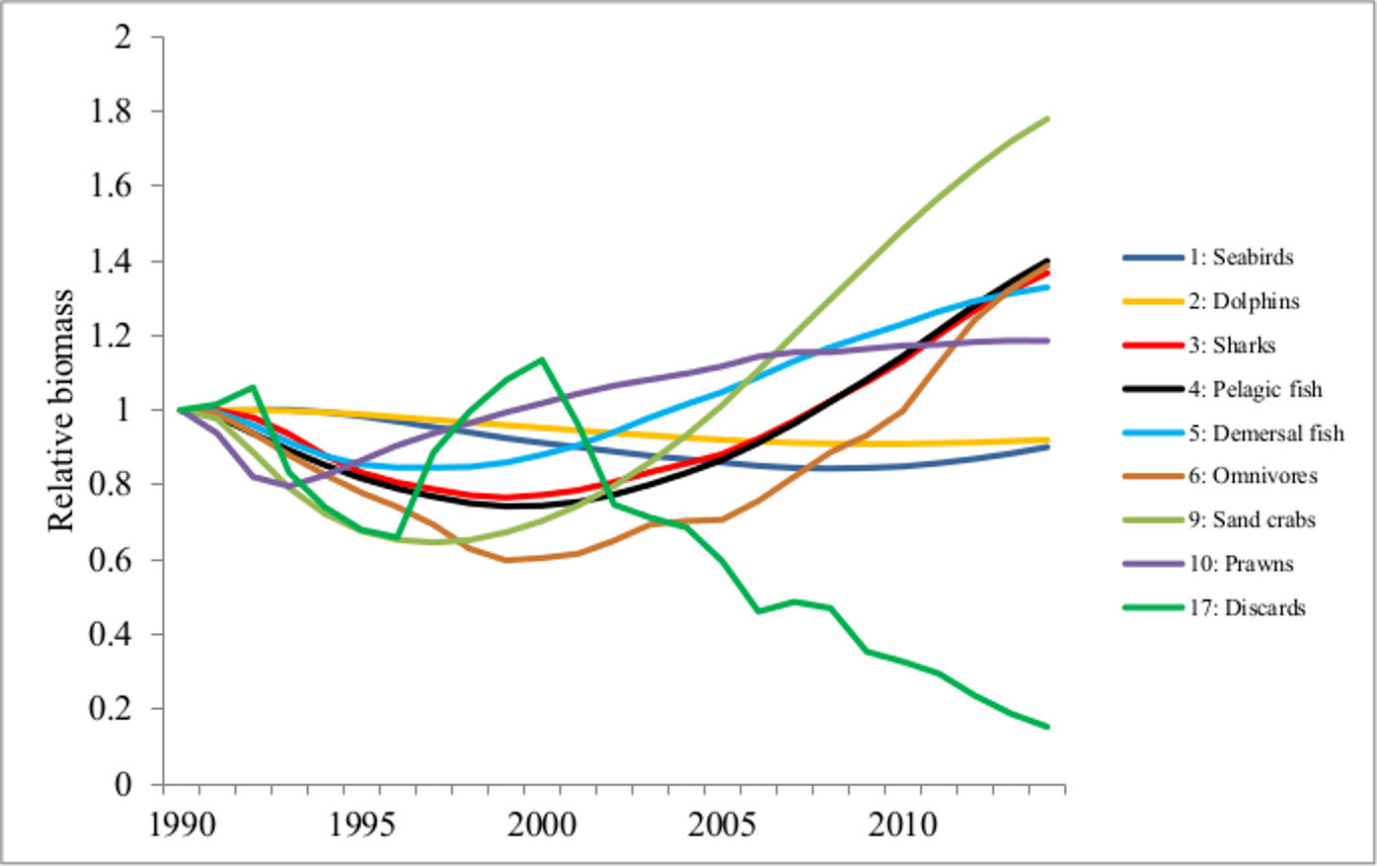
Changes in relative biomass of some groups in Moreton Bay ecosystem model in Scenario 1 –gradual reduction of PAFS.

**Fig 2:**
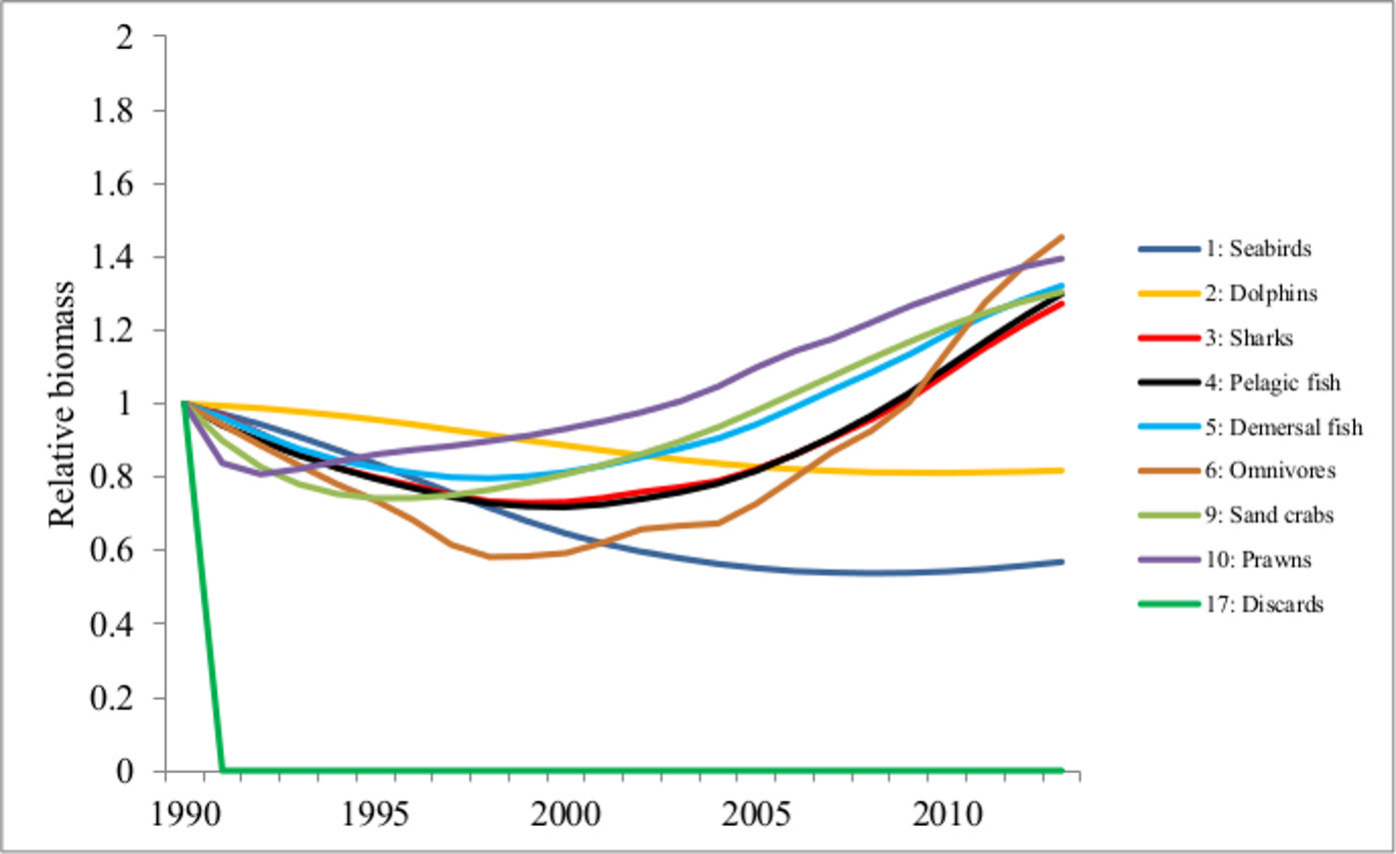
Changes in relative biomass of some groups in Moreton Bay ecosystem model in Scenario 2- ban on PAFS.

PAFS may increase the survival, reproduction and alter the social behaviour(s) of predators [3]. Under adverse and harsh environments, individuals able to exploit PAFS may still reproduce and survive with success [18] e.g. rats dwelling in subsidized habitats grew better than those in non-subsidized habitats when subjected to the same levels of environmental stress [54]. Studies have shown the importance of PAFS in supplementing energy requirements where natural food resources are scarce [7–9, 12]. In our study, for both scenarios there was an overall increase in the relative biomass of most functional groups over the study period, except for seabirds and dolphins. Seabirds and dolphins are the first and major beneficiaries of discards [30] as shown in the diet matrix (Table E in S2 File). In scenario 1 (where the discards were reduced gradually over time), the relative biomass of the major opportunistic species (seabirds and dolphins) was maintained, not declining drastically with the reduction of discards as expected. These species responded slowly to the reduction of PAFS (Fig 1). In contrast, their relative biomasses dropped (by between 3–37% for seabirds and 2–11% for dolphins) in scenario 2 where there was an abrupt ban on PAFS (Fig 2). The Mann-Whitney U test showed a statistically significant change in the biomass of dolphins (Z = -3.918; p<0.05) and seabirds (Z = 4.248; p<0.05) between the two scenarios. These results suggest that in the presence of PAFS, opportunistic species (seabirds and dolphins) are able to habituate better to the gradual reduction of PAFS than under their sudden removal. This could indicate that these opportunistic species are resilient to changes in food resources and may provide support for the hypothesis that PAFS increase the resilience of opportunistic species.

### 3) PAFS modify predator–prey interactions

**Fig 3:**
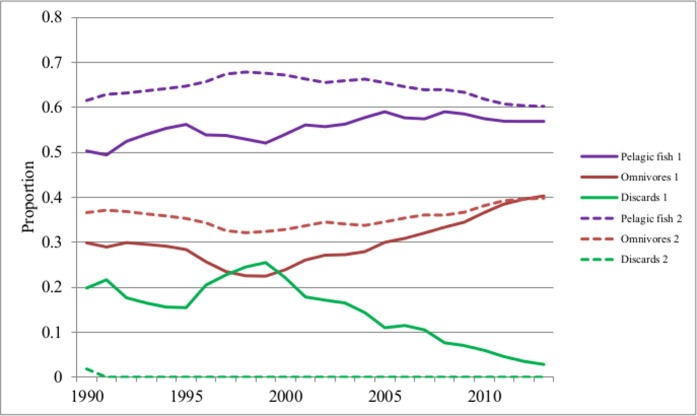
Changes in the proportions of the prey in the diet of seabirds from 1990 to 2013 (Solid lines = Scenario 1; dotted lines = Scenario 2).

Predator-prey interactions play a key role in ecosystem structuring [5, 55] and availability of PAFS result in changes in these interactions [3]. The results of the dynamic model showed that the major opportunistic species (seabirds and dolphins) exhibited prey switching when the discards were gradually reduced (Scenario 1) from consuming discards to mainly eating lower level omnivores; but when PAFS are banned abruptly (Scenario 2), opportunistic species exert greater pressure on their natural prey. For example, seabirds change their diet to increase the proportion of lower level omnivores, with only a slight increase in the major prey pelagic fish in their diet, in response to the deficit resulting from the gradual reduction of discards (Fig 3). Dolphins also show a similar response to gradual reduction of discards (Fig B in S1 File). The Mann-Whitney U tests on the proportions of prey in the diets of seabirds and dolphins for the two scenarios were significantly different (Table K in S2 File). These results on the changes in proportions of prey in the diets of the major (upper trophic level) opportunistic species illustrate how discards may alter predator-prey interactions and give support to the findings of studies reviewed by Oro *et al* [2]. These interactions become important as consumers previously subsidised by PAFS may switch their diet to a focus on specific prey species; if the species subsequently influenced are particularly important in the community the effects could be far-reaching [5]. The predatory opportunists (seabirds, dolphins and sharks) may potentially exert notable effects through top-down control over the complexity and structure of trophic interactions [5].

### PAFS and ecosystem stability

The results of the FCI (Fig C in S1 File) show that, in scenario 1, the FCI declined slightly from 1 to 0.86 between 1990 and 1992 and then increased gradually from 1992 to 2009 (to a maximum of 1.6) as the discards were being reduced. The FCI then dropped drastically to zero corresponding with the point when the discards reached a relative biomass of 0.368 (in 2009 from Fig 1). In scenario 2, the FCI dropped immediately after the ban reaching zero in 1992 (Fig C in S1 File). The Mann-Whitney U test showed a statistically significant difference between the FCI in the two scenarios (Z = 4.237; p<0.05). These results provide support for the findings of studies reviewed by Oro *et al* [2] that PAFS influence ecological processes such as ecosystem resilience and stability. The trend observed for FCI in scenario 1 suggests that the Moreton Bay ecosystem was initially stressed (between 1990 and 1992) then started to improve, maintaining its structure and slowly maturing as the discards were being reduced. The system reverted back to a stressed condition when the discards reach a certain minimal point. This suggests that a complete ban on the discards may stress the system and that the presence of a certain quantity of discards in the system might actually be good for the maintenance of the system’s structure and maturity, possibly because of the long exposure of the system to discards from fishing.

## Conclusions

Food subsidies have the potential to modify ecosystems. The concern over the effects of PAFS on ecosystem dynamics has led several jurisdictions to reduce or eradicate PAFS. However, these actions may have detrimental effects on some key ecological processes and impact negatively on species or communities that have commercial or conservation value; in some cases a ban or reduction of PAFS may be unnecessary [56]. The current study has shown that, in general, a gradual removal of PAFS may be beneficial by allowing species to habituate to food scarcity. The rate at which PAFS are removed will depend on the ecosystem in question and requires negotiation with major stakeholders (in case of this study, the fishing industry) and managers. Multiple hypothetical scenarios could be generated to explore different rates of PAFS reduction but here we have used empirical data from a real world scenario and the results could be useful in exploring these hypothetical scenarios.

An abrupt ban can have a negative impact on the scavenger species especially in ecosystems that have experienced provision of PAFS over a long period of time. As it has been demonstrated, PAFS may modify predator- prey interactions and influence ecosystem stability. When PAFS are removed completely from the system, under either scenario, ecosystem stability drops with the cycling index reaching zero, an indication of a stressed system. Clearly, after so many generations of exposure to the ready food supply of PAFS, a certain minimal level is required to maintain stability. However further studies are required to determine this level in different ecosystems as the effects of providing resource subsidies to higher trophic levels may differ depending on the type of subsidy and the species present [33]. Gradual removal of PAFS may be beneficial to allow species exploiting PAFS to switch gradually to alternative prey (and to avoid the predators from drastically reducing the prey populations and driving them to local extinction). Under these circumstances, a gradual reduction of PAFS as opposed to a sudden ban is recommended, and may be a useful approach in the management of PAFS.

Policy decisions can have important consequences for the management and conservation of natural ecosystems. Both the gradual removal and complete ban on PAFS may affect the ecosystem, decreasing the survival of scavenger species. Managers dealing with PAFS should consider the direct effects of PAFS removal on any endangered species within the system (such as seabirds, dolphins and sharks) that are of regional and global importance. Further studies are needed to determine whether PAFS should be removed completely, reduced or maintained at current levels, for different types of ecosystem, in order to provide guidance to managers and conservation agencies [7,13,17].

## Supporting Information

S1 File Supporting information Figures. (PDF)

S2 File Supporting information Tables and Text. (PDF)
