# Supplementary material for: Banning Fisheries Discards Abruptly Has a Negative Impact on the Population Dynamics of Charismatic Marine Megafauna
Source: PLoS One. 2015 Dec 11;10(12):e0144543. doi: 10.1371/journal.pone.0144543 (PMC4676608; doi:10.1371/journal.pone.0144543)
Supplement: S1 File — (PDF) [file pone.0144543.s001.pdf]

S1 File: Supporting information Figures

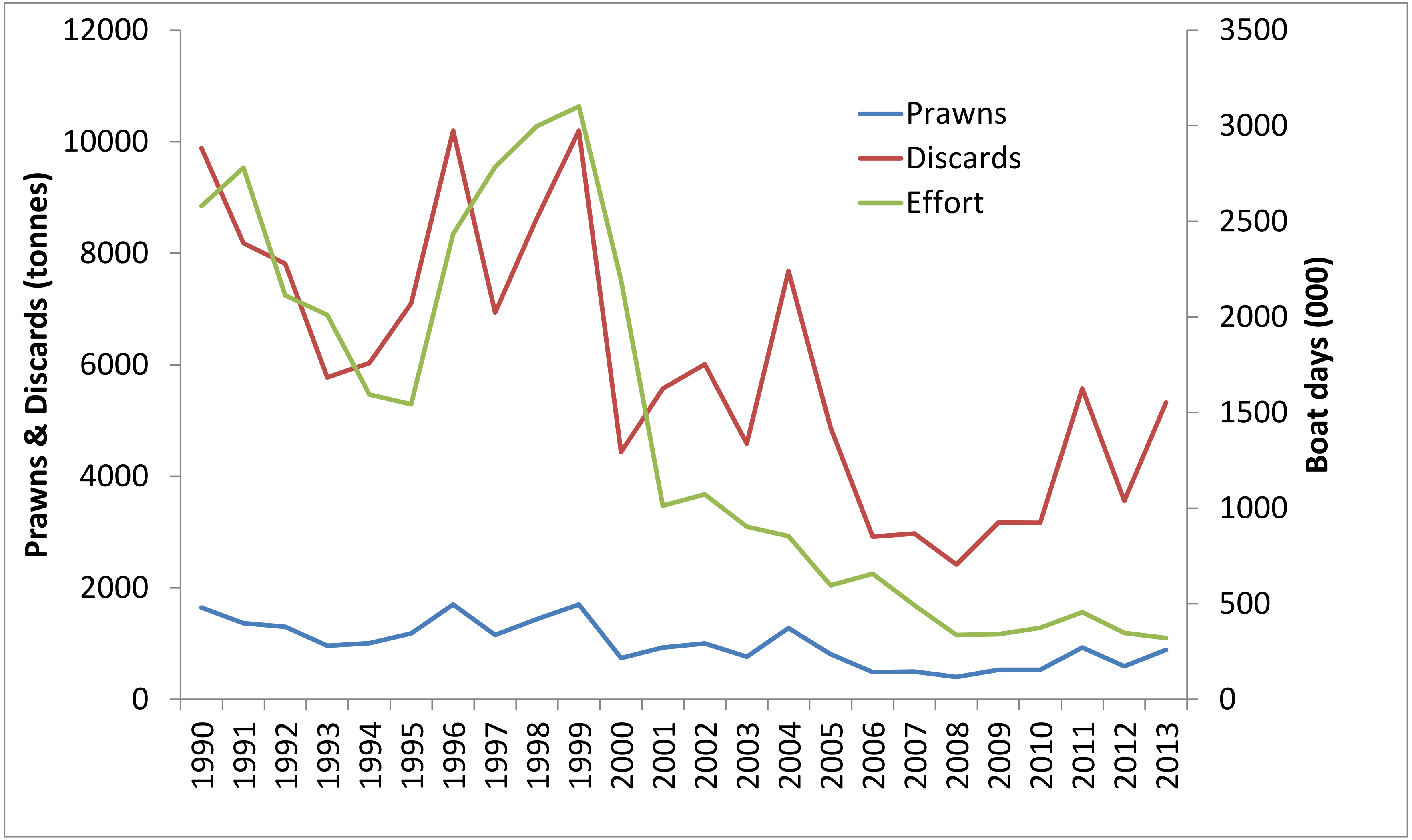

Fig A: Prawn catch, effort and discards from prawn trawling in Moreton Bay.
Data from Qfish database and the discards are estimated from prawn catches as by-catch data was not recorded.

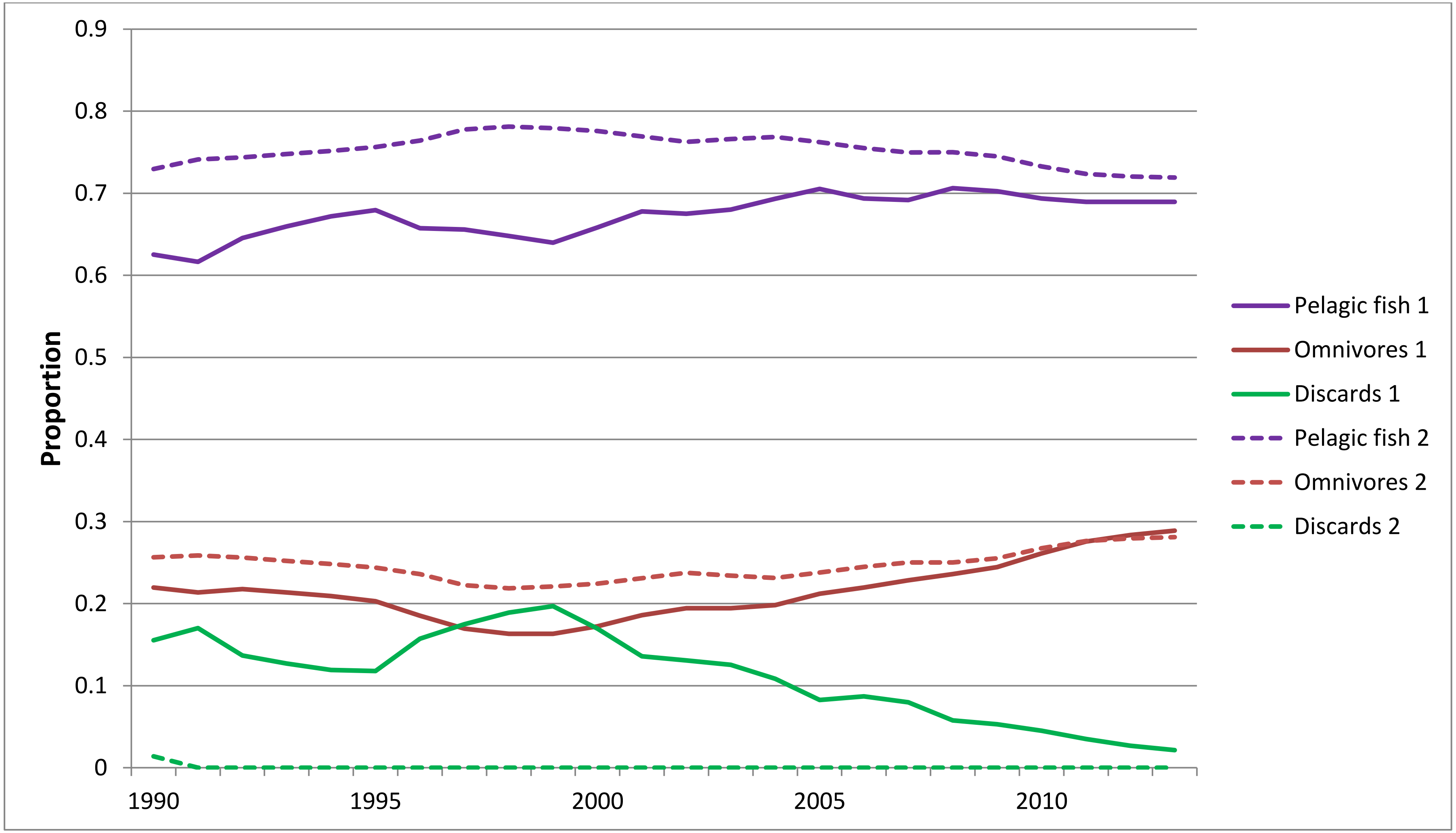

Fig B: Changes in the proportions of the prey in the diet of dolphins from 1990 to 2013
Solid lines – Scenario 1 and dotted lines- Scenario 2

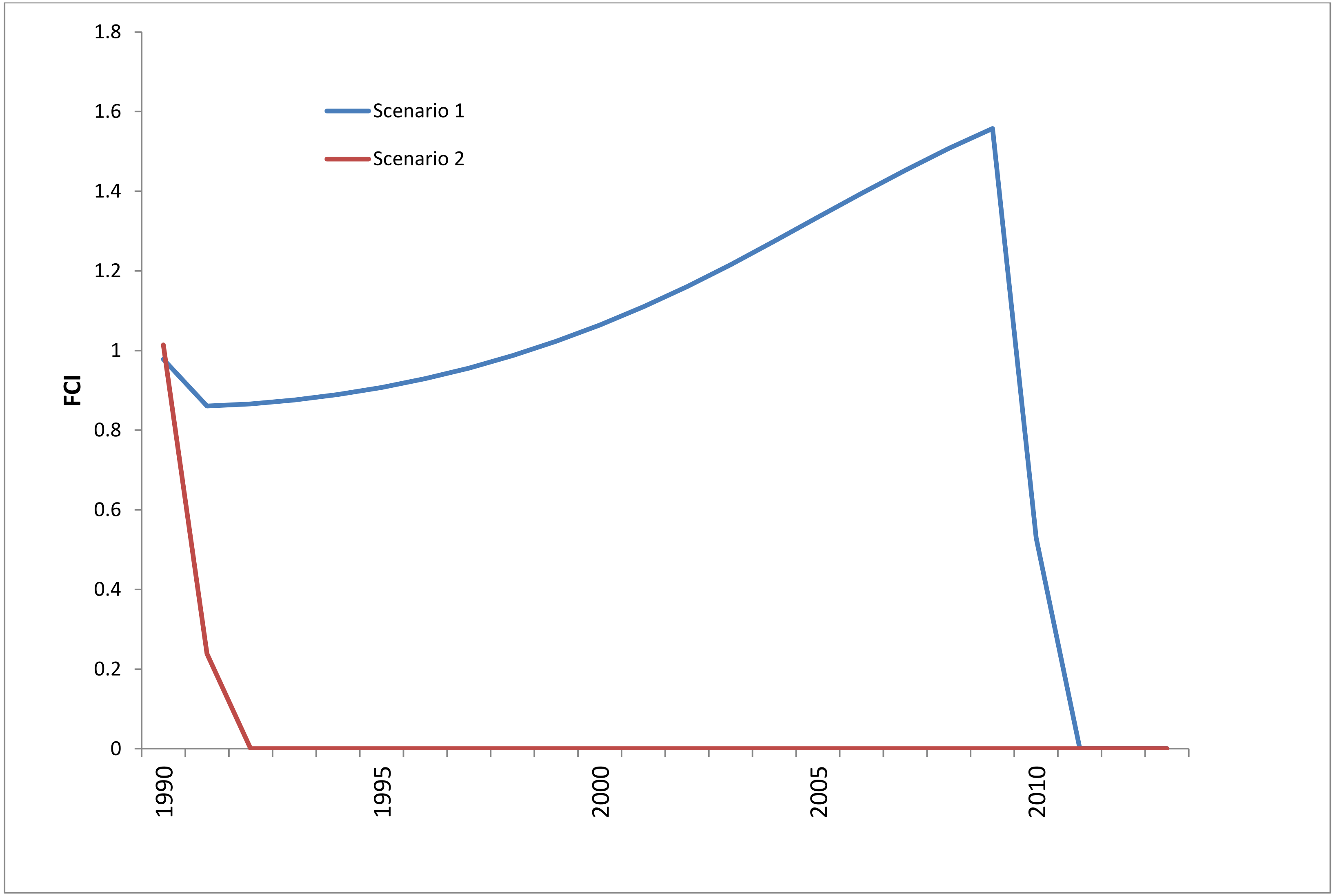

Fig C: Changes in Finn's Cycling Index (FCI) for the two Scenarios from 1990 to 2013
