# Supplementary material for: Banning Fisheries Discards Abruptly Has a Negative Impact on the Population Dynamics of Charismatic Marine Megafauna
Source: PLoS One. 2015 Dec 11;10(12):e0144543. doi: 10.1371/journal.pone.0144543 (PMC4676608; doi:10.1371/journal.pone.0144543)
Supplement: S2 File — (PDF) [file pone.0144543.s002.pdf]

S2 File: Supporting Information Tables and Text

Table A: Composition of discards from prawn trawling in Moreton Bay (Wassenberg and Hill, 1989)

**Group**	**Percentage** (weight)
Crustaceans	52
Echinoderms	18
Elasmobranchs	15
Teleost fish	8
Cephalopods	3
Others	4

Table B: Functional groups of the Moreton Bay ecosystem model

No.	Functional group	Components
1	Sea birds	Silver gulls, cormorants, crested terns*
2	Dolphins	Indo-Pacific bottlenose dolphins
3	Sharks	*Carcharhinus* spp
4	Pelagic fish	Trevally, Tuna, Mackerel and Whiting
5	Demersal fish	Rays, Cod, Bream, Snapper, Tailor and Flathead
6	Omnivores	Mullet and *Siganus* spp
7	Dugongs	*Dugong dugon*
8	Turtles	Green turtles
9	Sand crabs	*Portunus armatus*
10	Prawns	Penaeid prawns
11	Jellyfish	Scyphozoa, *Catostylus mosaicus*
12	Macrobenthos	Other crabs, mantis shrimps, annelids, gastropods, echinoderms
13	Zooplankton	Zooplankton
14	Seagrasses	Seagrasses
15	Macroalgae	Macroalgae
16	Phytoplankton	Phytoplankton
17	Discards	Discards
18	Detritus	Detritus

* (shorebirds (Skilleter pers. comm.) but included here based on the data sources which grouped them as seabirds)

Table C: Data sources for the different functional groups

No.	Functional group	Source
1	Sea birds	(Blaber and Wassenberg, 1989); Dunning (2007); Australia Fisheries Management Authority www.afma.gova.au/); (Wassenberg and Hill, 1990)
2	Dolphins	(Ansmann et al., 2012, Ansmann Ina Christiane, 2011); (Chilvers et al., 2003); Corkeron et al. (1997); (Chilvers and Corkeron, 2001, Chilvers et al., 2005)
3	Sharks	DAFF; (Stephen, 2007) (Taylor and Bennett, 2013); FishBase; Taylor, (Pierce et al., 2011)
4	Pelagic fish	DAFF; FishBase; (Kruck et al., 2009)
5	Demersal fish	DAFF, (Pierce et al., 2011, Kyne and Bennett, 2002); (Morton et al., 1987); FishBase; (Pollock and Williams, 1983); (Pillans, 2006); (Pollock and Williams, 1983, Pollock, 1982a, Pollock, 1982b, Morton et al., 1987
6	Omnivores	Gribble 2003; (Edgar and Shaw, 1995); (Capper et al., 2006); (Budarf et al., 2011)
7	Dugongs	Lanyon (Lanyon et al., 2010) (2003); Edgar and Shaw, (1995); Takahashi (2008); (Preen, 1995); (Chilvers et al., 2005); (Marsh et al., 1982); (Lanyon et al., 2010); (Perry et al., 1996); (Heinsohn et al., 1977); (Heinsohn et al., 1978); (Sheppard et al., 2006); Burgessa et al 2012; Marsh et al 1999
8	Turtles	Gribble 2003; Takahashi (2008); Limpus 2008; (Limpus et al., 1994); (Brand-Gardner et al., 1999); (Arthur et al., 2007); (Kuiper-Linley et al., 2007); (Arthur et al., 2008); (Bjorndal et al., 2000); (Chaloupka et al., 2004); Bjorndal (1997); (Chaloupka, 2001, Chaloupka et al., 2004, Chaloupka and Limpus, 2001)
9	Sand crabs	Courtney et al (2009); Criales-Hernandez et al 2006; DAFF; Williams, 1982; (Hill and Wassenberg, 1990); Weng 1992; Williams 1981; Wu & Shin 1997; Edgar 1990; (Campbell and Sumpton, 2009)
10	Prawns	Courtney et al (2009); Courtney et al (1995); Gribble 2003; DAFF; (Barber and Lee, 1975); (Masel and Smallwood, 2000); (Skilleter et al., 2005); Brey (2001)
11	Jellyfish	Wang 2012; Pitt & Kingsford, 2003(Pitt and Lucas, 2014); (Matt, 2007); (West et al., 2009); (Titelman et al., 2006); (Pitt and Kingsford, 2003b, Pitt and Kingsford, 2003a); Pitt et al 2007; (Pitt et al., 2007); (Peach and Pitt, 2005); (Kingsford et al., 2000); (Gershwin et al., 2010)
12	Macrobenthos	Edgar & Shaw (1995); Groenewold & Fonds (2000); SeaLifeBase, Brey (2001)
13	Zooplankton	Gribble 2003; Schlacher et al 2009; (Jacoby and Greenwood, 1989); (Greenwood, 1981); (Carr and Pitt, 2008); (Barber and Lee, 1975); (Greenwood, 1982)
14	Seagrasses	EPA 2007; (Young and Kirkman, 1975); (Kirkman, 1978); (Finn et al., 2010); (Boström et al., 2006); Takahashi et al 2008; (McMahon, 2003); (Peterken and Conacher, 1997); (Roelfsema et al., 2009); (Skilleter et al., 2007); (Perry et al., 1996); (Saunders et al., 2013); (Melville and Connolly, 2005)
15	Macroalgae	Fulton & Smith 2010; (Watkinson et al., 2005); (Pittman and Pittman, 2005); (Bell and Elmetri, 2007); (Arthur et al., 1991); Quigg et al 2008

16	Phytoplankton	Glibert (2006); (Wulff et al., 2011); (Gabric et al., 1998); (James et al., 1998); O'Donohue et al 2000; (Glibert et al., 2006, Glibert and Dennison, 2000); (Schlacher et al., 2008), (Quigg et al., 2010);
17	Discards	Gribble 2003; Wassenberg and Hill, 1990; Wassenberg and Hill 1989;
18	Detritus	Gribble 2003

Table D: Basic parameter estimates for Moreton Bay ecosystem model with discards (MB 1) (Values in italics were estimated by the model) TL= Trophic level; B= Biomass; P/B= Production/Biomass ratio; Q/B= Consumption/Biomass ratio; EE= Ecotrophic efficiency; P/Q= Production/Consumption ratio

	**Group name**	**TL**	**B (t/km^2)**	**P/B (/year)**	**Q/B (/year)**	**EE**	**P/Q**
1	Seabirds	3.79	0.0051	0.05	0.183	*0.196*	*0.274*
2	Dolphins	3.96	0.0918	*0.013*	0.045	*0*	0.3
3	Sharks	4.26	0.0366	0.225	1	*0.253*	*0.225*
4	Pelagic fish	3.5	0.43	0.4	1.4	*0.343*	*0.286*
5	Demersal fish	3.44	0.39	0.4	1.369	*0.955*	*0.292*
6	Omnivores	2.75	0.2	0.35	1.396	*0.958*	*0.251*
7	Dugongs	2	0.0075	0.009	0.030	*0*	*0.295*
8	Turtles	2	0.007	0.02	0.08	*0.786*	*0.25*
9	Sand crabs	2.90	0.6	0.7	2.4	*0.495*	*0.292*
10	Prawns	2.12	0.7	1.972	7.5	*0.368*	*0.263*
11	Jellyfish	3.11	1.075	0.09	0.4	*0.697*	*0.225*
12	Macrobenthos	2.45	0.8	1.8	6	*0.576*	*0.3*
13	Zooplankton	2.11	6.42	8.48	30	*0.42*	*0.283*
14	Seagrass	1	16	25.55	0	*0.001*	
15	Macroalgae	1	25.91	20	0	*0.001*	
16	Phytoplankton	1	11.3	28	0	*0.549*	
17	Discards	1	2			*0.960*	
18	Detritus	1	3.836			*0.006*	

Table E: Diet matrix for the Moreton Bay model with discards (MB1)

	Prey \ predator	1	2	3	4	5	6	7	8	9	10	11	12	13
1	Seabirds													
2	Dolphins													
3	Sharks													
4	Pelagic fish	0.508	0.63	0.562	0.04		0.002							
5	Demersal fish			0.246	0.121	0.089	0.016							
6	Omnivores	0.3	0.22				0.045							
7	Dugongs													
8	Turtles													
9	Sand crabs			0.08	0.1	0.23								
10	Prawns			0.092		0.381	0.022			0.14				
11	Jellyfish				0.112									
12	Macrobenthos				0.005	0.249	0.338			0.36			0.005	
13	Zooplankton				0.6		0.105			0.2	0.105	1	0.4	0.1
14	Seagrass						0.1	0.8	0.427				0.1	
15	Macroalgae				0.015		0.216	0.2	0.373				0.1	
16	Phytoplankton						0.04						0.095	0.9
17	Discards	0.192	0.15	0.02	0.007	0.001				0.018			0.01	
18	Detritus					0.05	0.116			0.282	0.745		0.29	
19	Import						0		0.2		0.15			
20	Sum	1	1	1	1	1	1	1	1	1	1	1	1	1

Table F: Basic parameter estimates for Moreton Bay ecosystem model without discards (MB 2) (Values in italics were estimated by the model) TL= Trophic level; B= Biomass; P/B= Production/Biomass ratio; Q/B= Consumption/Biomass ratio; EE= Ecotrophic efficiency; P/Q= Production/Consumption ratio

	**Group name**	**TL**	**B (t/km^2)**	**P/B (/year)**	**Q/B (/year)**	**EE**	**P/Q**
1	Seabirds	4.28	0.0051	0.05	0.183	*0*	*0.274*
2	Dolphins	4.34	0.0918	*0.013*	0.045	*0*	0.3
3	Sharks	4.32	0.0366	0.225	1	*0.247*	*0.225*
4	Pelagic fish	3.51	0.43	0.4	1.4	*0.352*	*0.286*
5	Demersal fish	3.44	0.39	0.4	1.369	*0.920*	*0.292*
6	Omnivores	2.75	0.2	0.35	1.396	*0.944*	*0.251*
7	Dugongs	2	0.00752	0.009	0.030	*0*	*0.295*
8	Turtles	2	0.007	0.02	0.08	*0*	*0.25*
9	Sand crabs	2.91	0.6	0.7	2.4	*0.447*	*0.292*
10	Prawns	2.12	0.7	1.972	7.5	*0.368*	*0.263*
11	Jellyfish	3.11	1.075	0.09	0.4	*0.697*	*0.225*
12	Macrobenthos	2.45	0.8	1.8	6	*0.544*	*0.3*
13	Zooplankton	2.11	6.42	8.48	30	*0.42*	*0.283*
14	Seagrass	1	16	25.55	0	*0.001*	
15	Macroalgae	1	25.91	20	0	*0.001*	
16	Phytoplankton	1	11.3	28	0	*0.549*	
17	Detritus	1	3.836			*0.006*	

Table G: Diet matrix for the Moreton Bay model without discards (MB2)

	Prey \ predator	1	2	3	4	5	6	7	8	9	10	11	12	13
1	Seabirds													
2	Dolphins													
3	Sharks													
4	Pelagic fish	0.7	0.78	0.582	0.04		0.002							
5	Demersal fish			0.246	0.121	0.089	0.016							
6	Omnivores	0.3	0.22				0.045							
7	Dugongs													
8	Turtles													
9	Sand crabs			0.08	0.1	0.23								
10	Prawns			0.092		0.381	0.022			0.14				
11	Jellyfish				0.112									
12	Macrobenthos				0.012	0.249	0.338			0.364			0.005	
13	Zooplankton				0.6		0.105			0.2	0.105	1	0.4	0.1
14	Seagrass						0.1	0.8	0.427	0.005			0.1	
15	Macroalgae				0.015		0.216	0.2	0.373	0.009			0.1	
16	Phytoplankton						0.04						0.095	0.9
17	Detritus					0.051	0.116			0.282	0.745		0.3	
18	Import								0.2		0.15			
19	Sum	1	1	1	1	1	1	1	1	1	1	1	1	1

Table H: Catch (t/km^2) data inputs for 1990 to 2013 derived from the Qfish database

Name	Sharks	Pelagic fish	Demersal fish	Omnivores	Sand crabs	Prawns	Beam trawl	Otter trawl	Line	Net	Pot
Pool code	3	4	5	6	9	10	1	2	3	4	5
Type	6	6	6	6	6	6	3	3	3	3	3
1990	0.0016	0.0108	0.0147	0.0533	0.0022	0.0941	1	1	1	1	1
1991	0.001	0.0118	0.0131	0.0414	0.0023	0.0779	1.251	1.0736	0.5584	1.1738	1.2895
1992	0.0012	0.0124	0.0146	0.0550	0.0017	0.0744	0.739	0.8210	0.2173	1.1170	1.2435
1993	0.0016	0.0153	0.0119	0.0287	0.0020	0.0550	0.4559	0.7876	0.3837	1.3303	1.9511
1994	0.0019	0.0129	0.0130	0.0433	0.0025	0.0575	0.8205	0.6127	0.1707	1.2914	2.4778
1995	0.0018	0.0132	0.0144	0.0543	0.0021	0.0676	0.7176	0.5951	0.1424	1.4930	2.7015
1996	0.0017	0.0154	0.0106	0.0523	0.0022	0.0971	0.4099	0.9573	0.1594	1.6563	2.4507
1997	0.0029	0.0156	0.0136	0.0343	0.0022	0.0661		1.1069	0.5003	2.0542	2.4768
1998	0.0025	0.0125	0.0117	0.0526	0.0025	0.0822		1.192	0.4611	1.6034	2.4541
1999	0.0026	0.0108	0.0131	0.0650	0.0029	0.0971		1.2328	0.5409	1.7484	3.4257
2000	0.0034	0.0163	0.0166	0.038	0.0028	0.0422	0.6456	0.8552	0.23	1.5498	3.5674
2001	0.0028	0.0135	0.0193	0.0736	0.0042	0.0531	1.8930	0.3545	0.1722	1.4349	3.7211
2002	0.0024	0.0084	0.0126	0.0397	0.003	0.0572	1.7667	0.381	0.1870	1.3605	2.8256
2003	0.0033	0.0139	0.0143	0.0479	0.003	0.0436	1.9327	0.31	0.4415	1.7329	3.2071
2004	0.0023	0.0100	0.018	0.0544	0.003	0.0731	1.4833	0.3013	0.3826	1.435	2.2847
2005	0.0024	0.0088	0.0179	0.0453	0.0025	0.0464	1.2356	0.2054	0.5067	1.1615	1.9007
2006	0.0029	0.0087	0.0141	0.0587	0.0024	0.0278	1.4861	0.2231	0.6186	1.1041	1.7981
2007	0.0030	0.0090	0.0174	0.0275	0.0022	2.83E-02	1.6593	0.1533	0.5023	0.9929	1.3933
2008	0.0028	0.0077	0.0141	0.0425	0.0021	0.0230	1.1978	0.1034	0.4843	1.1384	1.7488
2009	0.0019	0.0071	0.0102	0.0396	0.0029	0.0302	1.204	0.105	0.3795	0.6891	1.6108
2010	0.0012	0.0073	0.0077	0.0345	0.0022	0.0301	0.9408	0.1245	0.2579	0.5279	1.5161
2011	0.002	0.0074	0.0096	0.0311	0.0021	0.0531	0.421	0.1704	0.3191	0.5648	1.5425
2012	0.002	0.009	0.0099	0.0365	0.0025	0.0339	0.3557	0.1291	0.2173	0.631	1.5458
2013	0.002	0.0068	0.0094	0.049	0.0025	0.0507	0.2263	0.1218	0.2627	0.5807	1.7329

Table I: Biomass estimates from the Monte Carlo routine for CV = 0.1 to 0.5 on 100 trials

			CV=0.1		CV=0.2		CV=0.3		CV=0.4		CV=0.5	
	Group name	Mean	Lower limit	Upper limit	Lower limit	Upper limit	Lower limit	Upper limit	Lower limit	Upper limit	Lower limit	Upper limit
1	Seabirds	0.0051	0.00408	0.00612	0.00306	0.00714	0.00204	0.00816	0.00102	0.00918	0.0026	0.0102
2	Dolphins	0.0918	0.07344	0.11016	0.05508	0.12852	0.03672	0.14688	0.01836	0.16524	0.0459	0.1836
3	Sharks	0.0366	0.02928	0.04392	0.02196	0.05124	0.01464	0.05856	0.00732	0.06588	0.0183	0.0732
4	Pelagic fish	0.43	0.344	0.516	0.258	0.602	0.172	0.688	0.086	0.774	0.215	0.86
5	Demersal fish	0.39	0.312	0.468	0.234	0.546	0.156	0.624	0.078	0.702	0.195	0.78
6	Omnivores	0.2	0.16	0.24	0.12	0.28	0.08	0.32	0.04	0.36	0.1	0.4
7	Dugongs	0.00752	0.00602	0.00902	0.00451	0.01053	0.00301	0.01203	0.00150	0.01354	0.0038	0.0150
8	Turtles	0.007	0.0056	0.0084	0.0042	0.0098	0.0028	0.0112	0.0014	0.0126	0.0035	0.014
9	Sand crabs	0.6	0.48	0.72	0.36	0.84	0.24	0.96	0.12	1.08	0.3	1.2
10	Prawns	0.7	0.56	0.84	0.42	0.98	0.28	1.12	0.14	1.26	0.35	1.4
11	Jellyfish	1.075	0.86	1.29	0.645	1.505	0.43	1.72	0.215	1.935	0.5375	2.15
12	Macrobenthos	0.8	0.64	0.96	0.48	1.12	0.32	1.28	0.16	1.44	0.4	1.6
13	Zooplankton	6.42	5.136	7.704	3.852	8.988	2.568	10.272	1.284	11.556	3.21	12.84
14	Seagrass	16	12.8	19.2	9.6	22.4	6.4	25.6	3.2	28.8	8	32
15	Macroalgae	25.91	20.728	31.092	15.546	36.274	10.364	41.456	5.182	46.638	12.96	51.82
16	Phytoplankton	11.3	9.04	13.56	6.78	15.82	4.52	18.08	2.26	20.34	5.65	22.6

Table J: The number of paths for consumption and trophic levels (TL) for the groups in the models with discards (MB 1) and without discards (MB 2) from the Ecopath models

	**Functional group**	**MB 1**		**MB2**	
		**No. of pathways**	**TL**	**No. of pathways**	**TL**
1	Seabirds	119	3.79	112	4.18
2	Dolphins	119	3.96	107	4.34
3	Sharks	77	4.26	70	4.32
4	Pelagic fish	42	3.5	38	3.51
5	Demersal fish	21	3.44	19	3.44
6	Omnivores	76	2.75	69	2.75
7	Dugongs	2	2	2	2
8	Turtles	2	2	2	2
9	Sand crabs	11	2.9	11	2.91
10	Prawns	2	2.12	2	2.12
11	Jellyfish	1	3.11	1	3.11
12	Macrobenthos	6	2.45	5	2.45
13	Zooplankton	1	2.11	1	2.11

Table K: Mann-Whitney U tests for the prey biomass of (A) seabirds and (B) dolphins between the two scenarios

A: Seabirds

Prey	U-value	Z-score	p
Pelagic fish	0	-5.9282	0*
Omnivores	95	-3.9693	0.00008*
Discards	0	5.9282	0*

* Significant at p=0.05

B: Dolphins

Prey	U-value	Z-score	p
Pelagic fish	0	-5.9282	0*
Omnivores	114	-3.5775	0.00034*
Discards	0	5.9282	0*

*significant at p=0.05

Text A: Ecopath and Ecosim Equations (Christensen et al, 2008)

The Ecopath master equation defines the mass-balance between consumption, production, and net system exports over a given time period for each functional group (*i*) in an ecosystem as follows:

Production = Predation+ Fishery catches + Biomass accumulation + Net migration + other Mortality

$$B_i * (P/B_i)\, EE = Y_i + \sum B_j\, (Q/B)_j * DC_j$$

where *Bi* and *B j* are biomasses (the latter pertaining to *j*, the consumers of *i*); *P/Bi* is the ratio of production to biomass, equivalent to total mortality; *EEi* is the ecotrophic efficiency which is the fraction of production that is consumed within, or caught from the system (by definition between 0 and 1); *Y i* is equal to the fisheries catch (i.e., *Y* = *FB*); *Q/B j* is the food consumption per unit of biomass of j; and *DCji C* is the contribution of (*i*) to the diet of (*j*), and the sum is overall predators (*j*). Biomass accumulation and migration can also be added to the right hand side of the equation. Each model can deal with an unknown parameter (*B* or *EE*; *P/B* or *Q/B*) that can be estimated by the model, if no data is available.

The basics of Ecosim consist of biomass dynamics expressed through a series of coupled differential equations. The equations are derived from the Ecopath master equation and is as follows:

$$dB_i/dt = g_i \sum Q_{ji} - \sum Q_{ij} + I_i - (M0_i + F_i + e_i)B_i$$

where dBi/dt represents the growth rate during the time interval dt of group (*i*) in terms of its biomass, Bi, gi is the net growth efficiency (production/consumption ratio), *M0i* the non-predation ('other') natural mortality rate, Fi is fishing mortality rate, ei is emigration rate, Ii is immigration rate, (and $e_i \cdot B_i - I_i$ is the net migration rate). The two summations estimates consumption rates, the first expressing the total consumption by group (i), and the second the predation by all predators on the same group (i). The consumption rates, *Q*ji, are calculated based on the 'foraging arena' concept, where Bi's are divided into vulnerable and invulnerable components.

Text B: Model Calibration

The Ecosim model was calibrated using the varying vulnerability and forcing functions on primary production repeated for predator and prey/predator interactions as follows:

Baseline

Baseline and vulnerabilities

Baseline and primary production

Baseline and vulnerabilities and primary production

Fishing

Fishing and vulnerabilities

Fishing and primary production

Fishing and vulnerabilities and primary production

References for Table C

1. Blaber SJM, Wassenberg TJ (1989) Feeding ecology of the piscivorous birds Phalacrocorax varius, P. melanoleucos and Sterna bergii in Moreton Bay, Australia: diets and dependence on trawler discards. Marine Biology 101: 1-10.

2. Dunning J (2007) CRC Handbook of Avian Body Masses: CRC Press.

3. Wassenberg TJ, Hill BJ (1990) Partitioning of material discarded from prawn trawlers in Moreton Bay. Aust J Mar Freshwat Res 41: 27-36.

4. Ansmann IC, Parra GJ, Chilvers BL, Lanyon JM (2012) Dolphins restructure social system after reduction of commercial fisheries. Animal Behaviour 84: 575-581.

5. Ansmann IC (2011) Fine-Scale Population Structure of Indo-Pacific Bottlenose Dolphins, Tursiops aduncus, in Moreton Bay, Queensland, Australia: The University of Queensland, School of Biological Sciences

6. Chilvers BL, Corkeron PJ, Puotinen ML (2003) Influence of trawling on the behaviour and spatial distribution of Indo-Pacific bottlenose dolphins (Tursiops aduncus) in Moreton Bay, Australia. Canadian Journal of Zoology-Revue Canadienne De Zoologie 81: 1947-1955.

7. Chilvers BL, Corkeron PJ (2003) Abundance of Indo-Pacific bottlenose dolphins, Tursiops aduncus, Off Point Lookout, Queensland, Australia. Marine Mammal Science 19: 85-095.

8. Corkeron PJ (1997) Bottlenose dolphins, Tursiops truncatus, in south-east Queensland waters: social structures and conservation biology. In: Kemper. MHaC, editor. In Marine mammal research in the Southern Hemisphere 1: Status, ecology and medicine U.K.: Surrey Beatty and Sons, Chipping Norton,1–10.

9. Chilvers BL, Corkeron PJ (2001) Trawling and Bottlenose Dolphins' Social Structure. Proceedings: Biological Sciences 268: 1901-1905.

10. Chilvers BL, Lawler IR, Macknight F, Marsh H, Noad M, et al. (2005) Moreton Bay, Queensland, Australia: an example of the co-existence of significant marine mammal populations and large-scale coastal development. Biological Conservation 122: 559-571.

11. Stephen T (2007) Population Structure and Resource Partitioning among Carcharhiniform Sharks in Moreton Bay, Southeast Queensland, Australia: The University of Queensland, School of Biomedical Science PhD Thesis.

12. Taylor SM, Bennett MB (2013) Size, sex and seasonal patterns in the assemblage of Carcharhiniformes in a sub-tropical bay. Journal of Fish Biology 82: 228-241.

13. Taylor SM, Bennett MB (2008) Cephalopod dietary specialization and ontogenetic partitioning of the Australian weasel shark Hemigaleus australiensis White, Last & Compagno. Journal of Fish Biology 72: 917-936.

14. Pierce SJ, Scott-Holland TB, Bennett MB (2011) Community composition of elasmobranch fishes utilizing intertidal sand flats in moreton bay, Queensland, Australia. Pacific Science 65: 235-247.

15. Kruck NC, Chargulaf CA, Saint-Paul U, Tibbetts IR (2009) Early post-settlement habitat and diet shifts and the nursery function of tidepools during Sillago spp. recruitment in Moreton Bay, Australia. Marine Ecology Progress Series 384: 207-219.

16. Kyne PM, Bennett MB (2002) Diet of the eastern shovelnose ray, Aptychotrema rostrata (Shaw & Nodder, 1794), from Moreton Bay, Queensland, Australia. Marine and Freshwater Research 53: 679-686.

17. Morton RM, Pollock BR, Beumer JP (1987) The occurrence and diet of fishes in a tidal inlet to a saltmarsh in southern Moreton Bay, Queensland. Austral Ecology 12: 217-237.

18. Pollock BR, Williams MJ (1983) An assessment of the angling fishery for yellowfin bream, Acanthopagrus australis (Guenther), in Moreton Bay, Australia. Journal of Fish Biology 22: 125-132.

19. Pillans S (2006) Effectiveness of no-take Marine Reserves in Moreton Bay, subtropical Australia: University of Queensland, Centre for Marine Studies

20. Pollock BR (1982) Spawning period and growth of yellowfin bream, Acanthopagrus australis (Gunther), in Moreton Bay, Australia. Journal of Fish Biology 21: 349-355.

21. Pollock BR (1982) Movements and migrations of yellowfin bream, Acanthopagrus australis (Gunther), in Moreton Bay, Queensland, as determined by tag recoveries. Journal of Fish Biology 20: 245-252.

22. Gribble NA (2003) GBR-prawn: modelling ecosystem impacts of changes in fisheries management of the commercial prawn (shrimp) trawl fishery in the far northern Great Barrier Reef. Fisheries Research 65: 493-506.

23. Edgar GJ, Shaw C (1995) The production and trophic ecology of shallow-water fish assemblages in southern Australia III. General relationships between sediments, seagrasses, invertebrates and fishes. Journal of Experimental Marine Biology and Ecology 194: 107-131.

24. Capper A, Tibbetts IR, O'Neil JM, Shaw GR (2006) Feeding preference and deterrence in rabbitfish Siganus fuscescens for the cyanobacterium Lyngbya majuscula in Moreton Bay, south-east Queensland, Australia. Journal of Fish Biology 68: 1589-1609.

25. Budarf AC, Burfeind DD, Loh WKW, Tibbetts IR (2011) Identification of seagrasses in the gut of a marine herbivorous fish using DNA barcoding and visual inspection techniques. Journal of Fish Biology 79: 112-121.

26. Lanyon JM, Sneath HL, Long T, Bonde RK (2010) Physiological Response of Wild Dugongs (Dugong dugon) to Out-of-Water Sampling for Health Assessment. Aquatic Mammals 36: 46-46.

27. Lanyon JM (2003) Distribution and abundance of dugongs in Moreton Bay, Queensland, Australia. Wildlife Research 30: 397-409.

28. Edgar GJ, Shaw C (1995) The production and trophic ecology of shallow-water fish assemblages in southern Australia II. Diets of fishes and trophic relationships between fishes and benthos at Western Port, Victoria. Journal of Experimental Marine Biology and Ecology 194: 83-106.

29. Takahashi EM, Arthur KE, Shaw GR (2008) Occurrence of okadaic acid in the feeding grounds of dugongs (Dugong dugon) and green turtles (Chelonia mydas) in Moreton Bay, Australia. Harmful Algae 7: 430-437.

30. Preen A (1995) Diet of Dugongs: Are They Omnivores? Journal of Mammalogy 76: 163-171.

31. Marsh H, Channells PW, Heinsohn GE, Morrisey J (1982) Analysis of stomach contents of dugongs from Queensland. Australian Wildlife Research 9: 55-67.

32. Perry CJ, Dennison WC, Phillips RC, Walker DI, Kirman H (1996) Effects of dugong grazing on microbial processes in seagrass sediments. 371-371 p.

33. Heinsohn GE, Wake J, Marsh H, Spain AV (1977) The dugong (Dugong dugon (Müller)) in the seagrass system. Aquaculture 12: 235-248.

34. Heinsohn GE, Lear RJ, Bryden MM, Marsh H, Gardner BR (1978) Discovery of a Large Population of Dugongs off Brisbane, Australia. Environmental Conservation 5: 91-92.

35. Sheppard JK, Preen AR, Marsh H, Lawler IR, Whiting SD, et al. (2006) Movement heterogeneity of dugongs, Dugong dugon (Müller), over large spatial scales. Journal of Experimental Marine Biology and Ecology 334: 64-83.

36. Burgess EA, Lanyon JM, Brown JL, Blyde D, Keeley T (2012) Diagnosing pregnancy in free-ranging dugongs using fecal progesterone metabolite concentrations and body morphometrics: a population application. General and comparative endocrinology 177: 82-92.

37. Marsh H, Eros C, Corkeron P, Breen B (1999) A conservation strategy for dugongs: implications of Australian research. Marine & Freshwater Research 50: 979-990.

38. Limpus CJ, Couper PJ, Read MA (1994) The green turtle, Chelonia mydas, in Queensland: Population structure in a warm temperate feeding area. Memoirs of the Queensland Museum Brisbane 35: 139-154.

39. Brand-Gardner SJ, Lanyon JM, Limpus CJ (1999) Diet selection by immature green turtles, Chelonia mydas, in subtropical Moreton Bay, south-east Queensland. Australian Journal of Zoology 47: 181-191.

40. Arthur KE, O'Neil JM, Limpus CJ, Abernathy K, Marshall G (2007) Using animal-borne imaging to assess green turtle (Chelonia mydas) foraging ecology in Moreton Bay, Australia. Marine Technology Society Journal 41: 9-13.

41. Kuiper-Linley M, Johnson CR, Lanyon JM (2007) Effects of simulated green turtle regrazing on seagrass abundance, growth and nutritional status in Moreton Bay, south-east Queensland, Australia. Marine & Freshwater Research 58: 492-503.

42. Arthur KE, Boyle MC, Limpus CJ (2008) Ontogenetic changes in diet and habitat use in green sea turtle (Chelonia mydas) life history. Marine Ecology Progress Series 362: 303-311.

43. Bjorndal KA, Bolten AB, Chaloupka MY (2000) Green turtle somatic growth model: Evidence for density dependence. Ecological Applications 10: 269-282.

44. Chaloupka M, Limpus C, Miller J (2004) Green turtle somatic growth dynamics in a spatially disjunct Great Barrier Reef metapopulation. Coral Reefs 23: 325-335.

45. Chaloupka M (2001) A System-of-Equations Growth Function for Southern Great Barrier Reef Green Sea Turtles. Chelonian Conservation and Biology 4: 88-93.

46. Chaloupka M, Limpus C (2001) Trends in the abundance of sea turtles resident in southern Great Barrier Reef waters. Biological Conservation 102: 235-249.

47. Courtney AJ, Masel JM, Die DJ (1995) Temporal and spatial patterns in recruitment of three penaeid prawns in Moreton Bay, Queensland, Australia. Estuarine, Coastal and Shelf Science 41: 377-392.

48. Criales-Hernandez MI, Duarte LO, García CB, Manjarrés L (2006) Ecosystem impacts of the introduction of bycatch reduction devices in a tropical shrimp trawl fishery: Insights through simulation. Fisheries Research 77: 333-342.

49. Williams MJ (1982) Natural food and feeding in the commercial sand crab Portunus pelagicus Linnaeus, 1766 (Crustacea : Decapoda : Portunidae) in moreton bay, queensland. Journal of Experimental Marine Biology and Ecology 59: 165-176.

50. Hill BJ, Wassenberg TJ (1990) Fate of discards from prawn trawlers in Torres Strait. Aust J Mar Freshwat Res 41: 53-64.

51. Weng HT (1992) The sand crab (Portunus pelagicus (Linnaeus)) populations of two different environments in Queensland. Fisheries Research 13: 407-422.

52. Williams MJ (1981) Methods for analysis of natural diet in portunid crabs (Crustacea:Decapoda:Portunidae). Journal of experimental marine biology and ecology 52: 103-113.

53. Wu DX, Wang Y, Lin XP, Yang JY (2008) On the mechanism of the cyclonic circulation in the Gulf of Tonkin in the summer. Journal of Geophysical Research-Oceans 113.

54. Edgar GJ (1990) Predator-prey interactions in seagrass beds. II. Distribution and diet of the blue manna crab Portunus Pelagicus linnaeus at Cliff Head, Western Australia. Journal of Experimental Marine Biology and Ecology 139: 23-32.

55. Campbell MJ, Sumpton WD (2009) Ghost fishing in the pot fishery for blue swimmer crabs Portunus pelagicus in Queensland, Australia. Fisheries Research 95: 246-253.

56. Courtney AJ MK, S. Pascoe, M. F. O'Neill, G. M. Leigh, Y-G Wang, J. Innes1, M. Landers, M. Braccini, A. J. Prosser, P. Baxter and J. Larkin (2009) Harvest strategy evaluations and co-management for the Moreton Bay Trawl Fishery. . 204pp. p.

57. Kienzle M, Courtney AJ, O'Neill MF (2014) Environmental and fishing effects on the dynamics of brown tiger prawn (Penaeus esculentus) in Moreton Bay (Australia). Fisheries Research 155: 138-148.

58. Gribble NA, Wassenberg TJ, Burridge C (2007) Factors affecting the distribution of commercially exploited penaeid prawns (shrimp) (Decapod:Penaeidae) across the northern Great Barrier Reef, Australia. Fisheries Research 85: 174-185.

59. Barber WE, Lee CP (1975) Preliminary analysis of physical factors influencing the ingress of planktonic king prawn (Penaeus plebejus) postlarvae into Moreton Bay.

60. Masel JM, Smallwood DG (2000) Indications of Long Term Changes in the Species Composition and Catch Rates of Postlarval and Juvenile Prawns in Moreton Bay. Proceedings of the Royal Society of Queensland, The 109: 119-130.

61. Skilleter GA, Zharikov Y, Cameron B, McPhee DP (2005) Effects of harvesting callianassid (ghost) shrimps on subtropical benthic communities. Journal of Experimental Marine Biology and Ecology 320: 133-158.

62. Brey T (2001) The Virtual Handbook of Population dynamics. http://wwwthomas-breyde/science/virtualhandbook/navlog/indexhtml

63. Wang Y, Li SY, Duan LJ, Liu Y (2012) Fishery policy exploration in the Pearl River Estuary based on an Ecosim model. Ecological Modelling 230: 34-43.

64. Pitt KA, Kingsford MJ (2003) Temporal and spatial variation in recruitment and growth of medusae of the jellyfish, Catostylus mosaicus (Scyphozoa: Rhizostomeae). Marine and Freshwater Research 54: 117-125.

65. Pitt KA, Lucas CH (2014) Jellyfish blooms. Dordrecht: Springer Netherlands.

66. Matt H (2007) Jellies. Sea Power. Navy League of the United States 50 (6): 30.

67. West EJ, Welsh DT, Pitt KA (2009) Influence of decomposing jellyfish on the sediment oxygen demand and nutrient dynamics. Hydrobiologia 616: 151-160.

68. Titelman J, Riemann L, Sørnes TA, Nilsen T, Griekspoor P, et al. (2006) Turnover of dead jellyfish: Stimulation and retardation of microbial activity. Marine Ecology Progress Series 325: 43-58.

69. Pitt KA, Kingsford MJ (2003) Temporal variation in the virgin biomass of the edible jellyfish, Catostylus mosaicus (Scyphozoa, Rhizostomeae). Fisheries Research 63: 303-313.

70. Pitt KA, Kingsford MJ, Rissik D, Koop K (2007) Jellyfish modify the response of planktonic assemblages to nutrient pulses. Marine Ecology Progress Series 351: 1-13.

71. Peach MB, Pitt KA (2005) Morphology of the nematocysts of the medusae of two scyphozoans, Catostylus mosaicus and Phyllorhiza punctata (Rhizostomeae): implications for capture of prey. Invertebrate Biology 124: 98-108.

72. Kingsford MJ, Pitt KA, Gillanders BM (2000) Management of jellyfish fisheries, with special reference to the order Rhizostomeae. 85-156 p.

73. Gershwin LA, Zeidler W, Davie PJF (2010) Medusae (Cnidaria) of Moreton Bay, Queensland, Australia. Memoirs of the Queensland Museum 54: 47-108.

74. Groenewold S, Fonds M (2000) Effects on benthic scavengers of discards and damaged benthos produced by the beam-trawl fishery in the southern North Sea. Ices Journal of Marine Science 57: 1395-1406.

75. Schlacher TA, Connolly RM, Skillington AJ, Gaston TF (2009) Can export of organic matter from estuaries support zooplankton in nearshore, marine plumes? Aquatic Ecology 43: 383-393.

76. Jacoby CA, Greenwood JG (1989) Emergent zooplankton in Moreton Bay, Queensland, Australia - seasonal, lunar, and diel patterns in emergence and distribution with respect to substrata. Marine Ecology Progress Series 51: 131-154.

77. Greenwood JG (1981) Occurrences of congeneric pairs of Acartia and Pseudodiaptomus species (copepoda, calanoida) in Moreton Bay, Queensland. Estuarine, Coastal and Shelf Science 13: 591-596.

78. Carr EF, Pitt KA (2008) Behavioural responses of zooplankton to the presence of predatory jellyfish. Journal of Experimental Marine Biology and Ecology 354: 101-110.

79. Greenwood JG (1982) Dominance, frequency and species richness patterns in occurrences of Calanoid copepods in Moreton Bay, Queensland. Hydrobiologia 87: 217-227.

80. Young PC, Kirkman H (1975) The seagrass communities of Moreton Bay, Queensland. Aquatic Botany 1: 191-202.

81. Kirkman H (1978) Decline of seagrass in northern areas of Moreton Bay, Queensland. Aquatic Botany 5: 63-76.

82. Finn PG, Udy NS, Baltais SJ, Price K, Coles L (2010) Assessing the quality of seagrass data collected by community volunteers in Moreton Bay Marine Park, Australia. Environmental Conservation 37: 83-89.

83. Boström C, Jackson EL, Simenstad CA (2006) Seagrass landscapes and their effects on associated fauna: A review. Estuarine, Coastal and Shelf Science 68: 383-403.

84. McMahon K (2003) Population dynamics of Halophila ovalis after dugong grazing in a dynamic subtropical ecosystem. Gulf of Mexico Science 21: 122-122.

85. Peterken CJ, Conacher CA (1997) Seed germination and recolonisation of Zostera capricorni after grazing by dugongs. Aquatic Botany 59: 333-340.

86. Roelfsema CM, Phinn SR, Udy N, Maxwell P (2009) An Integrated Field and Remote Sensing Approach for Mapping Seagrass Cover, Moreton Bay, Australia. Journal of Spatial Science 54: 45-45.

87. Skilleter GA, Wegscheidl C, Lanyon JM (2007) Effects of grazing by a marine mega-herbivore on benthic assemblages in a subtropical seagrass bed. Marine Ecology Progress Series 351: 287-300.

88. Saunders MI, Leon J, Phinn SR, Callaghan DP, O'Brien KR, et al. (2013) Coastal retreat and improved water quality mitigate losses of seagrass from sea level rise. Global change biology 19: 2569-2583.

89. Melville AJ, Connolly RM (2005) Food webs supporting fish over subtropical mudflats are based on transported organic matter not in situ microalgae. Marine Biology 148: 363-371.

90. Lyons M, Phinn S, Roelfsema C (2011) Integrating Quickbird Multi-Spectral Satellite and Field Data: Mapping Bathymetry, Seagrass Cover, Seagrass Species and Change in Moreton Bay, Australia in 2004 and 2007. Remote Sensing 3: 42-64.

91. Watkinson AJ, O’Neil JM, Dennison WC (2005) Ecophysiology of the marine cyanobacterium, Lyngbya majuscula (Oscillatoriaceae) in Moreton Bay, Australia. Harmful Algae 4: 697-715.

92. Pittman SJ, Pittman KM (2005) Short-term consequences of a benthic cyanobacterial bloom (Lyngbya majuscula Gomont) for fish and penaeid prawns in Moreton Bay (Queensland, Australia). Estuarine, Coastal and Shelf Science 63: 619-632.

93. Bell PRF, Elmetri I (2007) Some chemical factors regulating the growth of Lyngbya majuscula in Moreton Bay, Australia: importance of sewage discharges. Hydrobiologia 592: 359-371.

94. Arthur KE, Limpus CJ, Balazs GH, Udy JW, Shaw GR (1991) Ecotoxicology of the cyanobacterium Lyngbya majuscula and the potential exposure of green turtles, Chelonia mydas, to tumour promoting compounds 46-46.

95. Quigg A, Litherland S, Phillips JA, Kevekordes K (2010) Phytoplankton productivity across Moreton Bay, Queensland, Australia: the impact of water quality, light and nutrients on spatial patterns. Memoirs of the Queensland Museum 54: 355-372.

96. Glibert PM, Heil CA, O'Neil JM, Dennison WC (2006) Nitrogen, Phosphorus, Silica, and Carbon in Moreton Bay, Queensland, Australia: Differential Limitation of Phytoplankton Biomass and Production. Estuaries and Coasts 29: 209-221.

97. Wulff F, Eyre BD, Johnstone R, Systemekologiska I, Stockholms U, et al. (2011) Nitrogen versus phosphorus limitation in a subtropical coastal embayment (Moreton Bay; Australia): Implications for management. Ecological Modelling 222: 120-130.

98. Gabric AJ, McEwan J, Bell PRF (1998) Water Quality and Phytoplankton Dynamics in Moreton Bay, South-Eastern Queensland I. Field Survey and Satellite Data. Mar Freshw Res 49: 215-215.

99. James M, Albert JG, Peter RFB (1998) Water quality and phytoplankton dynamics in Moreton Bay, south-eastern Queensland. II. Mathematical modelling. Marine & Freshwater Research 49: 227-239.

100. Glibert PM, Dennison WC (2000) Utilization of nitrogen and carbon by phytoplankton in Moreton Bay, Australia. Marine & Freshwater Research 51: 703-712.

101. Schlacher TA, Skillington AJ, Connolly RM, Robinson W, Gaston TF (2008) Coupling between Marine Plankton and Freshwater Flow in the Plumes off a Small Estuary. International Review of Hydrobiology 93: 641-658.

102. Wassenberg TJ, Hill BJ (1989) The effect of trawling and subsequent handling on the survival rates of the by-catch of prawn trawlers in Moreton Bay, Australia. Fisheries research 7: 99-110.

103. DAFF Department of Agriculture, Fisheries and Foresty. https://www.daf.qld.gov.au/ Accessed 29 August 2014

104. FishBase. Froese, R. and D. Pauly. Editors. 2015. FishBase. World Wide Web electronic publication. http://www.fishbase.org/ Accessed 4 May 2015
